# Legume Seeds as an Important Component of Human Diet

**DOI:** 10.3390/foods9121812

**Published:** 2020-12-07

**Authors:** Ryszard Amarowicz

**Affiliations:** Institute of Animal Reproduction and Food Research, Polish Academy of Sciences, 10-748 Olsztyn, Poland; r.amarowicz@pan.olsztyn.pl; Tel.:+48-895-234-627; Fax: +48-966-749-677

Legumes are an important source of nutrients (proteins, carbohydrates, water soluble vitamins, minerals) for human nutrition. They play important roles in chronic disease prevention. The beneficial effects of legumes are attributed to the presence of legume seed starch with a low glycemic index, dietary fiber (soluble and insoluble), several classes of phenolic compounds, and oligosaccharides [1]. Phenolic compounds of legumes possess strong antioxidant and antimicrobial activities. Oligosaccharides, acting as prebiotics, modify intestinal microbiota [1,2].

Some of the bioactive compounds present in legumes (e.g., trypisin inhibitors, condensed tannins, lectins, phytates) also exhibit anti-nutritional effects—decreased protein digestibility and availability of mineral compounds. Technological processes (non-thermal and thermal processing, hydrolysis, fractionation) can modify the functional properties (emulsifying activity and stability, foaming properties, water holding capacity) of legumes and legume products, as well as modify the activity of bioactive compounds present in legume seeds [1].

Grass pea (*Lathyrus sativus*) exhibits drought tolerance and thrives with minimal external inputs [3]. It is an ideal legume for resource-poor farmers from the Indian subcontinent, Ethiopia and in lesser extent North Africa, Australia, Asia, and Europe [4]. The contents of proteins, starch, lipids, mineral compounds, and energy in grass peas are similar to those of peas and faba bean [5]. Palmitic and linoleic acids are the main fatty acids of grass pea lipids [6]. From a toxicological point of view, the genetic and technological (soaking, cooking, cooking in boiled water at low or high pH) improvement of grass pea is very important for reducing the content of β-N-oxalyl-1-α,β-diamino-propionic acid (β-ODAP) [7]. This non-protein amino acid causes a neurolathyrism, a neurological disease of humans and domestic animals [8].

In the study of Rybiński et al. [9], the total phenolic compound contents of the of 30 varieties of grass pea ranged from 20.3 to 70.3 mg/100 g seeds. The seeds were characterized using Trolox equivalent antioxidant capacity values of 0.158–0.372 mmol Trolox/100 g seeds, and FRAP values of 0.487–1.189 Fe^2+^/100 g seeds. The total phenolics content of grass pea extract was correlated with the results of the FRAP (ferric-reducing antioxidant power) (r = 0.781) and ABTS (3-ethylbenzothiazoline-6-sulfonic acid) (r = 0.881) assays. The same correlation was observed between the results of both assays (r = 0.842). The authors concluded that grass pea seeds with reduced contents of β-ODAP after technological processing can be a source of phenolic compounds in a vegetarian or vegan diet, and in the general population. 

The fermentation of leguminous seeds reduces anti-nutritional compounds, improves protein and starch digestibility, reduces allergenicity, and increases seeds’ antioxidant capacity. Therefore, there is a growing interest in promoting the production of fermented leguminous seeds [10]. Tempeh is a traditional Indonesian food, produced by the fermentation of soybeans using *Rhizopus* species, having nutritional qualities and metabolic regulation functions [11].

Vital et al. [12] used white bean (*Phaseolus vilgaris* L.) as a material for tempeh preparation. The results indicated significant differences in the nutritional value of the tempeh produced from white bean and soybean. The produced tempeh samples did not present a risk of microbiological contamination for consumption. The white bean tempeh burgers showed similar appearances and a crispy consistency, but received lower scores for flavor, compared to the soybean burgers. The beany flavor present in white bean tempeh could be minimized by increasing the cooking time of the beans. According to authors, white bean tempeh can be a good alternative for healthy eating, and its manufacture could promote the production of new products made from beans. However, it is still necessary to improve the techniques of production and test new ingredients for the preparation of tempeh burgers to obtain higher acceptability.

Legume seeds are an important source of iron, zinc and copper for human. However Fe, Zn, and Cu absorption is reduced in the presence in seeds of such compounds as phytates and polyphenols, and especially tannins. Technologists recommend seed soaking and discarding the soaking water before cooking, but this leads to mineral loss.

The study of Feitosa et al. [13] aimed to evaluate iron, zinc and copper bioaccessibility in black beans cooked using a regular pan and pressure cooker, with and without the soaking water. The minerals were quantified by inductively coupled plasma mass spectrometry (ICP-MS). In addition, *myo*-inositol phosphates (InsP5, InsP6) were determined by HPLC, and total polyphenols and condensed tannins using colorimetric methods. Mineral bioaccessibility was determined by in vitro digestion and dialysis. All treatments resulted in a statistically significant reduction in total polyphenols (30%) and condensed tannins (20%). Only when discarding the soaking water was a loss of iron (6%) and copper (30%) observed, and InsP6 was slightly decreased (7%) in one treatment. The bioaccessibility values of Fe and Zn were low (about 0.2% iron and 35% zinc). A high bioaccessibility (about 70%) was obtained for cooper. Cooking beans under pressure without discarding the soaking water resulted in the highest bioaccessibility levels among all household procedures. Discarding the soaking water before cooking did not improve the nutritional quality of the beans.

Epicotyls from germinated soybeans (EGS) have great potential as sources of endogenous β-glucosidase. Furthermore, this enzyme may improve the conversion of isoflavones into their corresponding aglycones. This enzyme can also increase the release of aglycones from the cell wall of the plant materials, and epicotyls have been recommended as a potential industrial source of endogenous β-glucosidase [14].

The aim of the work of Yoshiara et al. [15] was to optimize the extraction of β-glucosidase from EGS. Next, the authors examined its application in defatted soybean cotyledon to improve the recovery of aglycones. The optimum extraction of β-glucosidase from EGS occurred at 30 °C and pH 5.0. Furthermore, the maximum recovery of aglycones (98.7%), which occurred at 35 °C and pH 7.0–7.6 during 144 h of germination, increased 8.5 times with respect to the lowest concentration. The higher bioaccessibilty of soybean isoflavone aglycones than that of glucosided was reported by several authors. Therefore, the results obtained may be useful for enhancing the benefits of soybean and soybean products and by-products.

The genus Prosopis is comprised of a group of nitrogen-fixing trees belonging to the Fabaceae family distributed in arid and semiarid regions of Asia, Africa, and America. The pod flour of Prosopis is a versatile ingredient with high potential for the food industry. It is rich in protein, sugars, and fiber, and is gluten-free [16]. One of the important representatives of the genus Prosopis is a legume tree mesquite (*Prosopis laevigata*) that is a widely distributed in Aridoamerica. In the work of Díaz-Batalla et al. [17], the nutritional value of mesquite seed flour and the effect of extrusion cooking on its bioactive components were assessed. The authors found mesquite seed flour to be a rich source of fiber (7.73 g/100 g) and protein (36.51 g/100 g). Valine was the only limiting amino acid of mesquite protein. The total phenolic compound contents in raw and extruded seed flour were 6.68 and 6.46 mg of gallic acid equivalents (GAE)/g, respectively. The high antioxidant potential of mesquite raw and extruded seed flour was confirmed using DPPH assay, and the values were 9.11 and 9.32 mg of ascorbic acid equivalents (AAE)/g, respectively. The extrusion did not generate Maillard reaction product (MRP). The authors found apigenin to be the only flavonoid in mesquite seed flour. This phenolic compound was stable in the extrusion. The authors concluded in their study that mesquite seed flour is a valuable plant food rich in good quality protein and active compounds. The extrusion cooking process of mesquite seed flour is an optional and versatile technology useful in the development of functional foods and the industrialization of this underutilized legume.

Peanut oil is derived from the peanut (*Arachis hypogaea*), a legume that is rich in proteins, vitamins, polyphenols, polyunsaturated fatty acids, and dietary fiber [18,19]. Highly refined peanut oil undergoes several industrial processes, including the extraction of protein allergen, discoloration through bleaching, and deodorization [20].

The study of Smithson et al. [21] reports the analysis of the purity of highly refined peanut oils (HRPO) that were adulterated either with vegetable oil (VO), canola oil (CO), or almond oil (AO) for food quality assurance purposes. It is a fast, simple, accurate, sensitive, and low-cost chemometric approach. The authors used the Fourier transform infrared (FTIR) spectra of the pure oils and adulterated HRPO samples for a partial-least-square (PLS) regression analysis. The obtained PLS regression figures-of-merit had very high linearity (R^2^ = 0.9942 or higher). The PLS regressions accurately determined the percentage compositions of adulterated HRPOs, with an overall root-mean-square relative percent error of 5.53%, and with the very low limit of detection of 0.02%. The developed PLS regressions continued to predict the compositions of newly prepared adulterated HRPOs over a period of two months, with incredible accuracy without the need for re-calibration. The protocol, due to its sensitivity, accuracy, and robustness, is potentially adoptable.
